# A LysR-Type Transcriptional Regulator Controls Multiple Phenotypes in *Acinetobacter baumannii*

**DOI:** 10.3389/fcimb.2021.778331

**Published:** 2021-11-04

**Authors:** Aimee R. P. Tierney, Chui Yoke Chin, David S. Weiss, Philip N. Rather

**Affiliations:** ^1^ Department of Microbiology and Immunology, Emory University School of Medicine, Atlanta, GA, United States; ^2^ Emory Vaccine Center, Atlanta, GA, United States; ^3^ Yerkes National Primate Research Center, Atlanta, GA, United States; ^4^ Division of Infectious Diseases, Department of Medicine, Emory University School of Medicine, Atlanta, GA, United States; ^5^ Emory Antibiotic Resistance Center, Atlanta, GA, United States; ^6^ Research Service, Department of Veterans Affairs, Atlanta Veterans Affairs (VA) Medical Center, Decatur, GA, United States

**Keywords:** *Acinetobacter baumannii*, AB5075, LysR-type transcriptional regulator, phenotypic heterogeneity, quorum sensing, motility, polysaccharide capsule, virulence

## Abstract

*Acinetobacter baumannii* is a multidrug-resistant, Gram-negative nosocomial pathogen that exhibits phenotypic heterogeneity resulting in virulent opaque (VIR-O) and avirulent translucent (AV-T) colony variants. Each variant has a distinct gene expression profile resulting in multiple phenotypic differences. Cells interconvert between the VIR-O and AV-T variants at high frequency under laboratory conditions, suggesting that the genetic mechanism underlying the phenotypic switch could be manipulated to attenuate virulence. Therefore, our group has focused on identifying and characterizing genes that regulate this switch, which led to the investigation of *ABUW_1132* (*1132*), a highly conserved gene predicted to encode a LysR-type transcriptional regulator. ABUW_1132 was shown to be a global regulator as the expression of 74 genes was altered ≥ 2-fold in an *1132* deletion mutant. The *1132* deletion also resulted in a 16-fold decrease in VIR-O to AV-T switching, loss of 3-OH-C_12_-HSL secretion, and reduced surface-associated motility. Further, the deletion of *1132* in the AV-T background caused elevated capsule production, which increased colony opacity and altered the typical avirulent phenotype of translucent cells. These findings distinguish *1132* as a global regulatory gene and advance our understanding of *A. baumannii*’s opacity-virulence switch.

## Introduction

The Gram-negative pathogen *Acinetobacter baumannii* poses a major threat to hospitalized patients. Cases of ventilator-associated pneumonia are among the most common *A. baumannii* infections, but incidences of skin and soft tissue infections, urinary tract infections, and sepsis are on the rise (Davis et al., 2005; Dijkshoorn et al., 2007; Peleg et al., 2008; Peleg and Hooper, 2010; Doyle et al., 2011; Weiner et al., 2016; Wong et al., 2017). Of primary concern is *A. baumannii*’s increasing resistance to treatment with antimicrobials, with 63% of infections caused by multidrug-resistant (MDR) strains (Clark et al., 2016; Lee et al., 2017; Centers for Disease Control and Prevention, 2019). In particular, *A. baumannii*’s rapidly growing resistance to carbapenem antibiotics and its ability to widely disseminate resistance *via* mobile genetic elements prompted the World Health Organization to name this organism as a critical priority for the research and development of new antimicrobial drugs in 2017 (World Health Organization, 2017). Further, its extreme resistance to desiccation and disinfectants makes it notoriously difficult to eradicate in hospital environments (Jawad et al., 1998; Chapartegui-Gonzalez et al., 2018; Rocha et al., 2018; Bravo et al., 2019; D’souza et al., 2019). In the face of such problematic phenotypes, an understanding of pathogenesis and virulence in this species is imperative. To meet this need, experiments conducted by our group have been carried out in the strain AB5075 (GenBank Accession Number CP008706.1), a highly virulent MDR clinical isolate that is genetically tractable (Jacobs et al., 2014; Gallagher et al., 2015).

**Figure 1:**
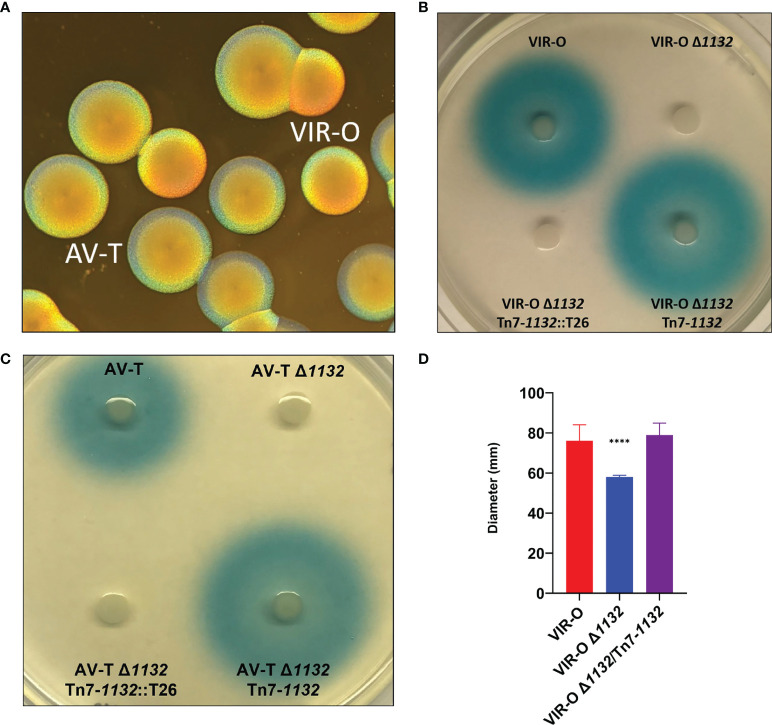
**(A)** AB5075 Wild-type opaque (VIR-O) and translucent (AV-T) colonies, viewed under a dissecting microscope with oblique lighting from underneath. **(B)** Qualitative assay of AHL secretion in which cultures of the wild-type (VIR-O), the VIR-O Δ*1132* mutant, the complemented mutant (VIR-O Δ*1132*/Tn7-*1132*), and a version of the complemented mutant disrupted by transposon insertion (VIR-O Δ*1132*/Tn7-*1132*::T26) were spotted onto a soft agar lawn containing X-Gal and an *Agrobacterium tumefaciens traG*::*lacZ* biosensor that reacts to the presence of exogenous AHL by cleaving X-Gal, forming a blue halo. **(C)** Qualitative assay of AHL secretion in the wild-type (AV-T), the AV-T Δ*1132* mutant, the complemented mutant (AV-T Δ*1132*/Tn7-*1132*), and a version of the complemented mutant disrupted by transposon insertion (AV-T Δ*1132*/Tn7-*1132*::T26). Signal secretion was analyzed as in panel **(B)**, except the amount of X-gal was increased 2-fold. **(D)** Surface-associated motility of wild-type (VIR-O), the VIR-O Δ*1132* mutant, and the complemented mutant (VIR-O Δ*1132*/Tn7-*1132*) measured on 0.3% Eiken Agar plates. A Welch’s ANOVA (****p < 0.00005) was carried out to assess **(D)** error bars indicate standard deviation of the mean.

Our group has sought to better understand the genetic mechanisms regulating *A. baumannii* virulence in light of our findings that clinical isolates of this species exhibit phenotypic heterogeneity resulting in virulent and avirulent colony opacity variants (Tipton et al., 2015; Chin et al., 2018). The virulent variant has a golden, opaque colony morphotype under oblique lighting and is termed VIR-O, while the avirulent variant is translucent and is termed AV-T (**Figure 1A**). In addition to differences in virulence, the VIR-O variant displays higher levels of capsule production, quorum sensing signal secretion, and surface-associated motility, while AV-T demonstrates greater production of biofilm and is able to utilize multiple carbon sources. Both variants switch back and forth at high frequency, with rates of approximately 4-13% conversion at 24 hours of colony growth and 20-40% at 48 hours (Chin et al., 2018). The two types have distinctly different genomic expression profiles as revealed by RNA sequencing, and a variety of gene products—including transcriptional regulators, a two-component system (OmpR/EnvZ), an efflux pump (ArpB), and a putative sRNA located upstream of a plasmid-encoded antibiotic resistance locus—appear to contribute to interconversion between VIR-O and AV-T (Tipton and Rather, 2016; Tipton et al., 2017; Chin et al., 2018; Anderson et al., 2020).

One such regulator, *ABUW_1645* (*1645*), is a TetR-type transcriptional regulator (TTTR) whose rate of expression is 150-fold higher in the AV-T state and whose overexpression in the VIR-O background drives conversion to the AV-T state (Chin et al., 2018). Although *1645* is crucial to the maintenance of the AV-T variant and is a key regulator of the VIR-O to AV-T switch, it does not appear to act in the same pathways as other previously discovered regulators of the switch such as *arpB* or *ompR* (Tipton and Rather, 2016; Tipton et al., 2017), a fact which emphasizes the complexity of the switching mechanism and the probable functional redundancy of many of the regulatory elements.

We continued to investigate additional genes to better understand the VIR-O and AV-T variants and the processes that regulate their interconversion. This led to the characterization of *ABUW_1132* (*1132*), a LysR-type transcriptional regulator (LTTR) that influences the VIR-O to AV-T switch. LTTRs are highly abundant in Proteobacteria (Reen et al., 2015) and are the most common type of transcriptional regulator in AB5075 at 24% (59/243) (Casella et al., 2017). They often function as global regulators and act in conjunction with a ligand molecule to repress and/or activate target genes (Maddocks and Oyston, 2008). Prototypical LTTRs are self-repressing and regulate gene(s) divergently transcribed from their own coding sequence, though these need not be the case.

A recent publication by our group detailed the identification of *1132* and its role in a *relA* mutant (*ΔrelA*), which exhibits increased quorum sensing signal secretion and hyper-motility (Perez-Varela et al., 2020). Quorum sensing in *A. baumannii* is carried out by a LuxI-LuxR type system composed of *abaI*, the autoinducer synthase, and *abaR*, the signal receptor and transcriptional regulator (Niu et al., 2008). We reported that the quorum sensing and motility phenotypes of *ΔrelA* were largely enacted through *1132*, which is overexpressed 14-fold in the absence of *relA* (Perez-Varela et al., 2020). Specifically, *1132* overexpression results in upregulation of the autoinducer synthase *abaI*, resulting in a large increase to secretion of the quorum sensing signal 3-OH-C_12_-homoserine lactone. Expression of the *abaR* transcriptional regulator is also increased, resulting in strong upregulation of one of AbaR’s targets: the *ABUW_3766-ABUW_3773* operon. This operon promotes production of the lipopeptide acinetin-505, which acts as a surfactant and gives rise to a hyper-motile phenotype.

This study builds on these findings and details multiple phenotypic changes resulting from the deletion of *1132;* including quorum sensing signal secretion, surface-associated motility, the virulence-opacity switch, capsule expression, and virulence in a mouse pneumonia model of infection.

## Results

### ABUW *1132* Is a Global Regulator

To identify genes and pathways regulated by *1132*, we carried out genome-wide transcriptional profiling by RNA sequencing of VIR-O Δ*1132* vs. wild-type VIR-O, which revealed a total of 74 differentially regulated genes in VIR-O Δ*1132* (greater than 2-fold change, p value less than 0.05) (**Supplementary Table 1**). These results showed that *1132* impacts transcription of a variety of genes involved in regulation, metabolism, protein synthesis, and possibly the cell stress response. Genes that encode ribosomal proteins, RNA polymerase, translation initiation factors, and both transcriptional and translational elongation factors are among genes that are upregulated in VIR-O Δ*1132*. On the other hand, several genes that are downregulated in the absence of *1132* encode a variety of enzymes involved in oxidative stress protection including catalases, peroxidases and others that interact with glutathione.

### Deletion of *1132* Impacts Quorum Sensing Signal Secretion and Motility

Deletion of *1132* results in loss of secretion of the quorum sensing signal 3-OH-C_12_-HSL (AHL) (**Figure 1B**). This is based on the inability to activate an *Agrobacterium tumefaciens traG*::*lacZ* fusion when grown on a soft agar lawn containing this biosensor strain and X-Gal (Niu et al., 2008; Paulk Tierney and Rather, 2019). We utilized a Tn7 transposon system (Ducas-Mowchun et al., 2019a) to provide single-copy complementation of *1132* (VIR-O Δ*1132*-Tn7/*1132*), which restored AHL secretion. This restoration is lost again if the Tn7 copy of *1132* is disrupted (**Figure 1B**). The loss of AHL secretion also occurs in the AV-T Δ*1132* mutant (**Figure 1C**).

Considering our previously published findings that *1132* overexpression activates the *abaI*-*abaR* system (Perez-Varela et al., 2020), we hypothesized that loss of AHL secretion was due to downregulation of *abaI* when *1132* is deleted. Surprisingly, our RNA sequencing analysis indicated wild-type levels of *abaI* expression in VIR-O Δ*1132*, which we confirmed by qRT-PCR (**Supplementary Figure 1**). This result suggested that the mutant cells synthesize AHL, but do not secrete it. To investigate this possibility, we conducted an assay utilizing the *A. tumefaciens* biosensor in which 10% SDS was added to a well at the center of the plate (**Supplementary Figure 2**). Cultures of wild-type VIR-O, an *abaI* mutant (VIR-O *abaI*::T26), and VIR-O Δ*1132* were then added to the plate in lines going toward the SDS-containing well. As expected, we saw that the wild-type VIR-O cells uniformly activate the biosensor. However, the VIR-O Δ*1132* cells show activation of the biosensor only at the point where the VIR-O Δ*1132* cells have been lysed by the SDS in the presence of non-lysed biosensor cells, which confirmed our hypothesis that AHL is synthesized but cannot exit the cell. Since there is no activation by an *abaI* mutant near the SDS, the activating signal in the *1132* mutant is 3-OH-C_12_-HSL and not a released metabolite. These results indicate a more complicated role for *1132* in quorum sensing than simple regulation of *abaI*, which is further considered in the *Discussion* section.

We previously reported that overexpression of *1132* increases surface-associated motility 3.8-fold and that this effect requires both *abaI* and the *ABUW_3766-ABUW_3773* operon (Perez-Varela et al., 2020). As expected, both VIR-O Δ*1132* and AV-T Δ*1132* demonstrate a significant decrease in motility compared to their wild-type counterparts, both which are complemented by the single-copy chromosomal insertion of *1132* (**Figure 1D**, only VIR-O results shown).

The *1132* deletion behaves in a manner opposite to *1132* overexpression with respect to motility, but the mechanism for this is unclear. As previously mentioned, *abaI* mRNA levels are unaffected by *1132* deletion, and although the RNA sequencing data shows a 1.5-fold downregulation of *abaR*, there are no transcriptional differences in the *ABUW_3766-ABUW_3773* operon (**Supplementary Table 1**). We also considered the possibility that AHL itself acts as a surfactant to some extent and that the loss of AHL secretion could reduce motility. To test this, we constructed a double mutant of *1132* and *abaR*—to control for quorum sensing-directed motility—and measured motility on 0.3% Eiken agar plates containing 1 μM synthetic 3-OH-C_12_-HSL. However, motility was similar between the solvent control (38.75 mm ± 0.83) and the plate with AHL added (41.0 ± 1.73) p = 0.11.

### Role of *1132* in Regulation of the VIR-O to AV-T Switch

**Figure 2:**
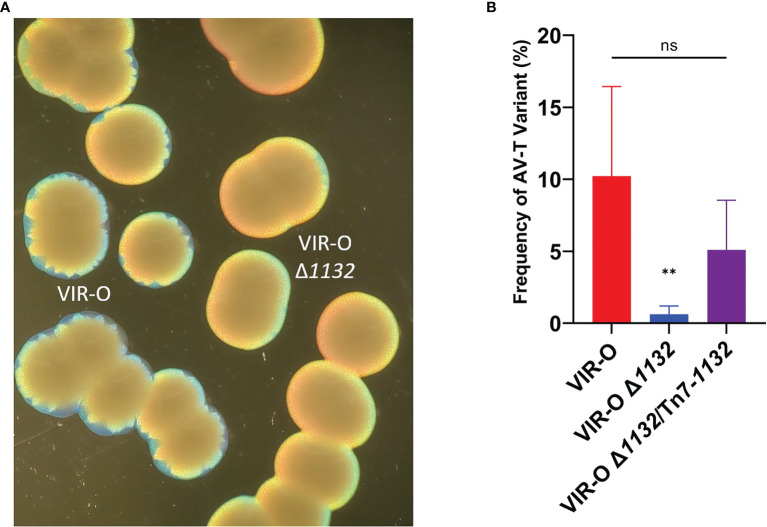
The Reduced Switching Phenotypes in VIR-O Δ*1132*. **(A)** Micrograph showing the loss of sectoring in VIR-O Δ*1132* colonies compared to wild-type VIR-O colonies at 24 hours of growth. **(B)** Quantification of switching frequencies demonstrating restoration of normal switching of VIR-O Δ*1132* through single copy complementation (VIR-O Δ*1132*/Tn7-*1132)*. The wild-type VIR-O and VIR-O Δ*1132* controls each have integrated an empty version of the pUC18T-mini-Tn7T-Apr-LAC insertion element into the attTn7 site. All micrographs were taken under a dissecting microscope illuminated from below the plate at an angle. All quantitative switching assays represent six colonies from each strain. A two-tailed Mann-Whitney test (**p < 0.005) was carried out for **(B)**, and error bars represent the standard deviation of the mean. ns, not significant. The p value represents a comparison of both wild-type to mutant and mutant to the complemented strain.

VIR-O Δ*1132* exhibited lower levels of colony sectoring than observed for the wild-type VIR-O on 0.5X LB agar (**Figure 2A**). This lack of sectoring indicated a decreased rate of switching to the AV-T variant, and we subsequently quantified a 16.4-fold decrease in the switching frequency (**Figure 2B**). To confirm these effects were due to *1132* deletion, we provided single-copy complementation of *1132* using the Tn7 transposon system. This strain exhibited the wild-type phenotype of colony sectoring and restored VIR-O to AV-T switching to wild-type levels (**Figure 2B**).

### Deletion of *1132* in the AV-T Background Increases Capsule

**Figure 3:**
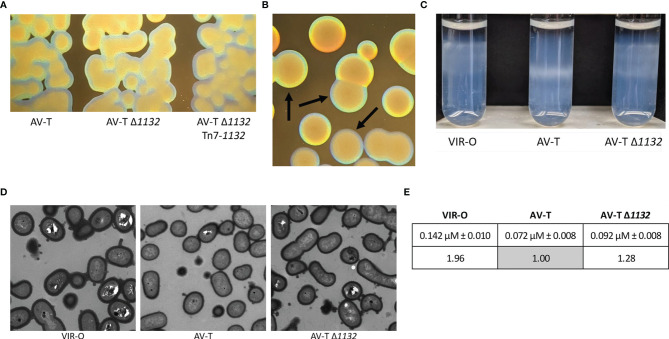
Deletion of *1132* in the AV-T background alters the opacity phenotype and capsule expression. **(A)** Micrographs comparing representative colonies of AV-T, AV-T Δ*1132* and AV-T Δ*1132* Tn7-*1132*. **(B)** Micrographs comparing colonies of AV-T Δ*1132*, indicated by arrows, to VIR-O Δ*1132*. Micrographs were taken under a dissecting scope lit from underneath at an angle. **(C)** Wild-type VIR-O, wild-type AV-T, and AV-T Δ*1132* cells were layered onto a Percoll gradient (top layer 40%, bottom layer 50%) and centrifuged for 30 minutes at 3,000 xg. AV-T Δ*1132* cells migrate between the stopping points for VIR-O and AV-T wild-types, indicating intermediate capsular levels with high levels of heterogeneity in the AV-T Δ*1132* mutant. Photograph shown is one of six experimental replicates. **(D)** Transmission electron micrographs of representative wild-type VIR-O, wild-type AV-T, and AV-T Δ*1132* cells, stained with Ruthenium red. **(E)** Averages ± standard deviation of the mean of capsule widths of these three strains converted to ratios to the wild-type AV-T. The difference between the wild-type AV-T and AV-T Δ*1132* capsule widths is significant at p < 0.0001 as determined by a Student’s two-tailed *t* test.

We further noted that AV-T Δ*1132* colonies are more opaque than wild-type AV-T colonies, although AV-T Δ*1132* is still translucent compared to the VIR-O Δ*1132* colonies (**Figures 3A, B**). Single-copy chromosomal complementation of the AV-T Δ*1132* mutant restored wild-type levels of translucence (**Figure 3A**). We first hypothesized that the increased opacity indicated hyper-switching from AV-T to VIR-O within the colony; however, the rate of switching was the same as the wild-type AV-T (**Supplementary Figure 3**). We then considered that the AV-T Δ*1132* mutant’s increased opacity may be due to increased levels of capsule.

Previous work revealed that wild-type VIR-O cells exhibit a 2-fold increase in capsule compared with the wild-type AV-T (Chin et al., 2018). We utilized a Percoll density gradient, a method that was recently described as a method to separate *A. baumannii* strains by capsule level (Kon et al., 2020), to compare capsular polysaccharide levels in AV-T Δ*1132* to wild-type VIR-O and AV-T cells. As seen in **Figure 3C**, the gradient is able to distinguish between capsule levels of the wild-type VIR-O and AV-T variants, with the AV-T cells migrating significantly further than the VIR-O cells. Intriguingly, AV-T Δ*1132* cells exhibited a high degree of heterogeneity and occupied a space in the gradient layer that was intermediate to that of the wild-type VIR-O and AV-T. We interpret this result as indication that deletion of *1132* causes a dysregulation and increase of capsule in the AV-T state, which imparts the more opaque appearance of AV-T Δ*1132*.

To confirm the effect of *1132* deletion on capsule, we next carried out TEM imaging of cell samples of wild-type VIR-O, wild-type AV-T, and AV-T Δ*1132* stained with Ruthenium red (**Figure 3D**). We used ImageJ to measure capsule width in 100 cells per strain with 3 measurements taken per cell, which revealed average capsule widths of 0.142 µM ± 0.010, 0.072 µM ± 0.008, and 0.092 µM ± 0.008 in VIR-O, AV-T, and AV-T Δ*1132*, respectively (**Figure 3E**). The resulting ratio of these values is 1.96:1.00:1.28, which demonstrates the 2-fold difference in capsule previously seen in the VIR-O vs. AV-T (Chin et al., 2018) and confirms a 28% increase in capsule in the AV-T background when *1132* is deleted. The difference between the wild-type AV-T and AV-T Δ*1132* is highly significant at p < 0.0001 as determined by a Student’s two-tailed *t* test.

In light of these results, we considered that, in the AV-T state, *1132* may regulate the K locus genes that encode the proteins largely responsible for the biosynthesis and export of capsular polysaccharide (CPS). We assessed representative genes from the K locus—*manB, galE*, *ABUW_3818, ABUW_3820, ABUW_3821, ABUW_3830*, *wza, wzb*, and *wzc*—by qRT-PCR across three sets of samples (**Supplementary Figure 4**). These results indicated that *1132* did not transcriptionally regulate the K locus genes.

### The Δ*1132* Mutation Increases Virulence in the AV-T Background

**Figure 4:**
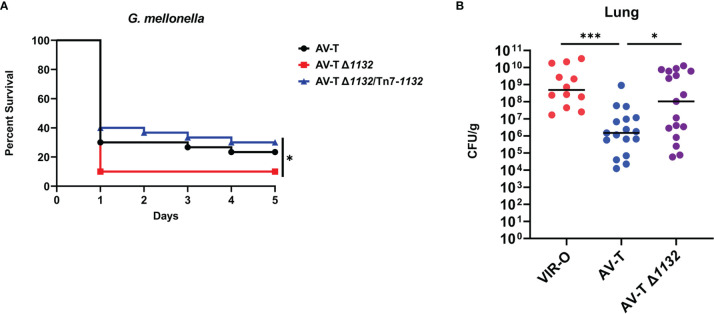
Deletion of *1132* in AV-T background increases virulence. **(A)** *Galleria mellonella* infected with AV-T Δ*1132* are killed at higher rates compared with wild-type AV-T or the complemented mutant (AV-T Δ*1132*/Tn7-*1132*). **(B)** Bacterial CFU/g recovered from mice lungs 24 hours after intranasal inoculation with pure cultures of wild-type VIR-O, wild-type AV-T, or AV-T Δ*1132*. A Log-Rank (Mantel-Cox) test (*p < 0.05) was carried out for **(A)** and a two-tailed Mann-Whitney test (*p < 0.05; ***p < 0.0005) was carried out for **(B)**.

Capsule is a known virulence factor in *A. baumannii*, and presumably contributes to virulent phenotype of the VIR-O variant relative to AV-T (Russo et al., 2010; Chin et al., 2018; Singh et al., 2018; Tipton et al., 2018; Talyansky et al., 2021). The intermediate capsular levels of AV-T Δ*1132* therefore suggested that this deletion may increase virulence. Before initiating virulence studies, we first tested the growth rates of the AV-T and AV-T Δ*1132* strains and found no significant differences under laboratory conditions (**Supplementary Figure 5A**. Using the *Galleria mellonella* (waxworm) model of infection, a modest, but statistically significant increase in AV-T Δ*1132* virulence was observed (**Figure 4A**). Waxworms injected with the wild-type AV-T control showed a 23.3% survival rate after five days, while only 10% of those injected with AV-T Δ*1132* survived. Complementation of AV-T Δ*1132* with the single-copy chromosomal insertion of *1132* (AV-T Δ*1132*/Tn7-*1132*) reversed the increase in virulence, bringing the rate of survival back up to 30%.

We next examined the virulence of AV-T Δ*1132* in a mouse pneumonia model of infection. In three experiments, mice were intranasally inoculated with 1 x 10^8^ CFU/mL of VIR-O (n=10), AV-T (n=17), or AV-T Δ*1132* (n=17). At 24 hours post-inoculation, lungs were harvested, and the CFU/g for each tissue was calculated where a significant increase in CFU/g in AV-T Δ*1132* was observed compared to wild-type AV-T in the lungs (**Figure 4B**). Both strains exhibited a similar number of VIR-O variants recovered from the lungs (approximately 0.1%). The highly virulent VIR-O variant was unaffected by the Δ*1132* mutation in both a *Galleria mellonella* waxworm model and in a mouse pneumonia model (**Supplementary Figures 5B, C**) and both strains exhibited similar growth rates *in-vitro* (**Supplementary Figure 5A**).

## Discussion

This study confirms 1132 as a global transcriptional regulator that impacts multiple pathways: surface-associated motility, AHL secretion, regulation of the opacity-virulence switch, and capsule expression. Most importantly, our work demonstrates that an *1132* deletion, and possibly its natural downregulation, has the potential to increase virulence of the typically avirulent translucent colony variant in the clinical isolate AB5075. The *1132* gene was highly conserved in all completed *A. baumannii* genome sequences (n = 330), where at least 98.7% nucleotide homology was observed. The gene was also conserved in *A. nosocomialis* (91.7% or greater nucleotide identity), *A. seifertii* (89.8% or greater identity) and *A. pittii* (83.7% or greater identity). Given the high conservation of *1132* among *A. baumannii* strains, it is likely that the *1132*-associated phenotypes we have reported here and in a previous publication (Perez-Varela et al., 2020) will also be conserved in other strains.

Some questions remain regarding the mechanisms through which 1132 acts in the described phenotypes. As previously mentioned, in our earlier publication we determined that overexpression of *1132* causes large increases in AHL secretion and surface-associated motility. This occurs through activation of the *abaI*-*abaR* system and follows a well-defined downstream pathway ending with overproduction of the surfactant acinetin-505 (Perez-Varela et al., 2020) Perplexingly, although the deletion of *1132* results in phenotypes opposite to that of *1132* overexpression—loss of both AHL secretion and motility—our experiments show that neither of these phenotypes are due to transcriptional downregulation of the *abaI*-*abaR* system or the *ABUW_3766-ABUW_3773* operon. A post-transcriptional effect of *1132* on *abaI* also seems unlikely, as VIR-O Δ*1132* cells lysed in the presence of the AHL-detecting biosensor are able to activate the *traG*::*lacZ* fusion, indicating a functional AbaI protein (**Supplementary Figure 2**). It is possible that the length of the *N*-acyl chain of the AHL molecule is altered due to the global changes that occur when *1132* is deleted. Both shortened and elongated chains could affect the hydrophobicity of the AHL molecule and therefore have the potential to disrupt diffusion or export of the autoinducer across the membrane. Due to the trapping of signal within the cell, it is possible that the quorum sensing response is altered, possibly being activated earlier in cell density, or being constitutive. With respect to capsule, the effect of 1132 on capsule thickness did not involve transcriptional changes in representative genes within the capsule locus. Therefore, the *1132* mutation may alter biosynthetic pathways that impact precursors for capsule synthesis in a manner that increases their abundance and results in enhanced capsule thickness.

This work revealed that *1132* positively regulated the VIR-O to AV-T switch and experiments to address the mechanism for this increase are ongoing. It is possible that one or more of several regulatory genes revealed by the RNA sequencing as differentially regulated by 1132 is involved. However, it is intriguing that other known regulators of the switch, including the OmpR/EnvZ two-component system and the ArpB efflux pump, were not regulated by 1132. A possibility is that the *1132* deletion creates a cellular metabolome that withholds ligands or other molecules required by other known regulators of the switch, such as the TetR regulator *ABUW_1645* (Chin et al., 2018). It is important to note that the lack of AHL secretion is not expected to impact the switch, as deletion of *abaI* does not cause alteration of switching frequency (Tipton et al., 2015).

Our work here has important implications for the characterizations of VIR-O cells as virulent and AV-T cells as avirulent. While the increase in virulence in the AV-T variant caused by *1132* deletion was modest, this finding demonstrates that stochastic downregulation of *1132*—potentially mediated by RelA’s repression of *1132*—may allow an AV-T cell to be moderately virulent in a mammalian host without necessitating a switch to the VIR-O state. We have previously hypothesized that AV-T cells possess an advantage in natural environments due to their versatility in utilization of carbon sources and their improved formation of biofilm (Chin et al., 2018). Combined, these observations suggest that AB5075, and possibly other *A. baumannii* strains, could survive in a natural environment while existing in a virulent state.

## Materials and Methods

### Bacterial Strains and Growth Conditions

All experiments were conducted using the clinical isolate AB5075. For all experiments, cultures were started from frozen glycerol stocks containing at least 99.5% of the desired colony variant (VIR-O or AV-T). Cultures were grown in either broth or on solid media containing LB (10 g tryptone, 5 g yeast extract, and 5 g NaCl per liter) and 1.5% agar (Difco) (“1X” LB agar) or 0.8% agar (“0.5X” LB agar).

### Methods to Distinguish Colony Variants

Cultures were plated or streaked on 0.5X LB agar and viewed under a dissecting microscope, illuminated from underneath at an oblique angle. Colonies must be viewed at high colony density (at least 100 colonies per plate) to effectively distinguish VIR-O and AV-T colonies.

### Detection of 3-OH-C_12_-HSL

Plates containing the *Agrobacterium tumefaciens traG*::*lacZ* biosensor and X-Gal were prepared as described in Paulk Tierney and Rather (2019). AB5075 cell cultures were grown in 2 mL LB to OD_600_ ~ 0.3, and 1 µL was spotted onto the soft agar lawn. Plates were incubated at 28°C overnight.

### Quantification of VIR-O and AV-T Switching

Dilutions were plated from frozen pure stocks of the strains to be assessed, and plates were incubated at 37°C for a set number of growth hours. High density plates were used to verify the purity of the stock. Isolated colonies were extracted from the plate by cutting out a section of agar underlying the colony and then resuspended and dilutions plated. The percent variant was then determined for each set of resuspended colonies from each strain. All experiments were performed twice with three colonies each for a total of six colonies.

### Electroporation

Cell cultures for competent cells were grown in 2 mL LB to OD_600_ ~ 0.5 (mid-log phase) at 37°C, shaking from frozen stocks. Cultures were pelleted and washed twice in sterile dH_2_O, then resuspended to accommodate a volume of approximately 50 µL of cell per electroporation. Plasmid minipreps or ligation products mixed with cells were added to 2 mm cuvettes and electroporated at 2.5 kV. Cells were recovered in 1 mL LB at 37°C, shaking for one hour, then plated onto media containing selective antibiotics.

### Construction of Deletion Mutations

In-frame deletion of *ABUW_1132* was generated using sucrose counterselection and a suicide vector containing the *sacB* marker (pEX18Tc) using methods previously described (Hoang et al., 1998). Briefly, PCR amplification (Phusion polymerase, Thermo-Fisher Scientific) was used to amplify the 2-kb regions upstream and downstream of the gene to be deleted, gel purified (UltraClean 15 DNA Purification Kit, MO BIO Laboratories), and ligated (Fast-Link DNA Ligation Kit, Epicentre Biotechnologies). The 4-kb ligation product was PCR amplified, gel purified, and ligated into the pEX18Tc vector MCS. The product vector was verified by sequencing and then transformed into competent *E. coli* Transformax EC100D cells (Epicentre Biotechnologies) by electroporation. The resulting suicide vector was confirmed by PCR and transformed by electroporation into AB5075, grown to OD_600_ of 0.5 in 2-mL LB and washed twice in 10% glycerol. Transformants were plated on 1X LB agar plus Tetracycline (5-µg/mL) to yield single-crossover mutants. Counterselection was carried out at room temperature on 1X LB plates with 10% sucrose and no NaCl, and colonies were screened by PCR for the deletion. Primer sequences are recorded in **Supplementary Table 2**.

To construct the Δ*1132*, *abaR*::T26 double mutant, genomic DNA was purified from an *abaR*::T26 mutant obtained from the University of Washington AB5075 transposon mutant library. Genomic DNA was then electroporated into VIR-O Δ*1132*. Transformants were selected on 1X LB plus Tetracycline (5-µg/mL) and confirmed by PCR.

### Construction of Single-Copy Complementation in Deletion Mutants

We utilized a Tn7-based single-copy insertion element system (Ducas-Mowchun et al., 2019a; Ducas-Mowchun et al., 2019b) to reintroduce *1132* into the VIR-O Δ*1132* and AV-T Δ*1132* deletion mutants. We opted to use a segment of *1132* that includes a large portion of the up and downstream regions (GenBank Accession NZ_CP008706.1:1152309-1156236). This is the portion of the genome contained within the original *1132-*containing fragment isolated from a high-copy AB5075 chromosomal library during the screen that led to our first discovery of *1132* (Perez-Varela et al., 2020), and previous experiments showed that this fragment allows for optimal expression of *1132*. A modified version of this construct containing a T26 transposon (tetracycline) in *1132* had also been made to confirm loss of the phenotypes presumably caused by *1132* overexpression. This was made by digestion of the plasmid with PmlI, which cuts once near the beginning of the *1132* ORF, and re-ligation with a PCR-amplified T26 transposon. The original *1132*-containing fragment and the modified version with *1132*::T26 were excised from these plasmids by digestion with XbaI, which flanks the site into which the library was cloned, and gel purified to be used in construction of the suicide vector.

The pUC18T-mini-Tn7T-Apr-LAC construct was digested with SpeI and ligated with the *1132*- or *1132*::T26-containing fragment (Fast-Link DNA Ligation Kit, Epicentre Biotechnologies). The ligation product was transformed into competent *E. coli* Transformax EC100D cells (Epicentre Biotechnologies) by electroporation. The resulting constructs—pUC18T-mini-Tn7T-Apr-LAC/*1132* and pUC18T-mini-Tn7T-Apr-LAC/*1132*::T26—were confirmed by PCR and sequencing then transformed into pure cultures of VIR-O Δ*1132* and AV-T Δ*1132* by electroporation along with the helper plasmid pTNS2. Transformant colonies were screened by PCR for insertion into the *att*Tn7 site and those containing the correct insertion were again verified by sequencing. Wild-type Tn7 and Δ*1132*-Tn7 control strains were also made in both VIR-O and AV-T backgrounds using the same methods with an empty pUC18T-mini-Tn7T-Apr-LAC construct. Primer sequences are recorded in **Supplementary Table 2**.

### RNA Preparation and qRT-PCR Analysis

Strains for qRT-PCR analysis in **Supplementary Figure 1** were prepared for RNA isolation by growing them from pure frozen stocks to OD_600_ ~ 0.15 then plating 100 µL on a 1X LB plate. Plates were incubated at 37°C for 6 hours, then pooled with 3 mL ice cold LB. Resuspensions were normalized to within OD_600_ of 0.01, then 1 mL of the resuspension was pelleted, flash frozen in an ethanol-dry ice bath and stored at -80°C. The resuspension cultures were streaked on 0.5X LB to ensure sample purity.

Strains for qRT-PCR analysis in **Supplementary Figure 4** were grown up to match the growth conditions of the cells prepared for electron microscopy (see below). Samples were grown from pure frozen stocks in overnight cultures of 2 mL LB and were then shaken at 37°C to the same OD_600_ of 0.3. 20 µL of each culture was then plated as a line onto a 0.5X plate, allowed to incubate at 37°C for 4 hours, and then harvested off the plate. Scraped cells were resuspended to an OD_600_ of 0.6 in 2 mL LB. 1 mL of cells was pelleted, flash frozen in an ethanol-dry ice bath, then stored at -80°C. The resuspension cultures were streaked on 0.5X LB to ensure sample purity.

RNA was prepared using the Epicentre MasterPure RNA Purification kit according to the manufacturer’s protocols. The resulting nucleic acid product was purified to remove DNA contamination using the Invitrogen TURBO DNA-*free* kit according to the manufacturer’s instructions. Following quantification of RNA concentration using a NanoDrop ND-1000 spectrophotometer, cDNA was prepared from 1 µg of RNA using the High-Capacity cDNA Reverse Transcription Kit by Applied Biosystems and subsequently diluted 1:10 in nuclease-free water. qRT-PCR experiments were carried out on a Bio-Rad CFX Connect Real-Time PCR Detection System using iQ SYBR Green Supermix reverse transcriptase from Bio-Rad. RNA purity was confirmed by qRT-PCR through the inclusion of template controls made without reverse transcriptase. qRT-PCR data was analyzed by the delta-delta Ct method (2^–∆∆Ct^) with comparison to *16S* as an internal control. This method was carried out for three biological replicates. Each biological replicate had three technical replicates for each primer set. Primer sequences are recorded in **Supplementary Table 2**.

### RNA Sequencing and Analysis

Three independent sets of RNA were prepared from each of AB5075 strains VIR-O and VIR-O Δ*1132*. Cultures were grown in 2 mL LB from pure frozen stocks to OD_600_ ~ 0.5 (shaking, 37°C) and were streaked on 0.5X LB to ensure purity of samples. 1 mL of cells was pelleted and flash frozen in a dry ice bath before being stored at -80°C. RNA was prepared using the Epicentre MasterPure RNA Purification kit according to the manufacturer’s protocols.

RNA quality control, sequencing, and analysis was carried out by the Yerkes Non-Human Primate Genomics Core at Emory University. RNA quantity and quality assessments were carried out using a Thermo Nanodrop2000 and Agilent 2100 Bioanalyzer, respectively. RNA sequencing was carried out using an Illumina HiSeq 3000, and reads were normalized and mapped using Cufflinks software. The RNA-Seq data discussed in this publication have been deposited in NCBI’s Gene Expression Omnibus and are accessible through GEO Series accession number GSE185730 (https://www.ncbi.nlm.nih.gov/geo/query/acc.cgi?acc=GSE185730).

### Percoll Density Gradient

Method slightly modified from Kon et al. (2020). Cell cultures of VIR-O, AV-T, and AV-T Δ*1132* were grown in 2 mL LB from pure frozen stocks to OD_600_ ~ 0.6 (shaking, 37°C). For each strain, 600 µL of cells was pelleted and washed in PBS 1X, then resuspended in 100 µL PBS 1X. Percoll solutions were prepared by first mixing 9 parts Percoll (Sigma Aldrich Cat. P4937) and 1 part 1.5 M NaCl, which was further diluted using 0.15 M NaCl to make 40% and 50% Percoll solutions. For each cell sample to be tested, 1 mL of 40% Percoll was added to a 12x75 mm glass tube (Fisherbrand 14958C), and 1 mL 50% Percoll was gently layered underneath using a 1cc syringe and a 1.5 in. 23G needle (BD 305194). 100 µL of cells were gently layered at the top of the gradient. Samples were spun at 3,000 xg for 30 minutes at room temperature and then visually assessed. This protocol was repeated 6 times.

### Electron Microscopy

Ruthenium red-lysine fixation and cell staining and subsequent transmission electron microscopy were performed by the Robert P. Apkarian Integrated Electron Microscopy Core at Emory University using previously described techniques (Fassel et al., 1997; Fassel et al.,1998; Beaussart et al., 2014). Samples were provided to the EM Core as follows: cells grown from pure frozen stocks in overnight cultures were shaken at 37°C until OD ~ 0.30, and 20 µL of each culture was plated as a line onto the same 0.5X LB plate. Plates were then incubated at 37°C for 4 hours and stored overnight at 4°C before being transported to the EM Core.

Capsule widths in the electron micrographs were measured by ImageJ 1.53e (Rasband, W.S., ImageJ, U. S. National Institutes of Health, Bethesda, Maryland, USA, https://imagej.nih.gov/ij/, 1997-2018). Three measurements were taken for 100 cells of each strain at the same magnification, which were averaged and then converted to micrometers.

### *Galleria mellonella* Larvae (Waxworm) Virulence Assays

Waxworms were purchased from Speedy Worm (www.speedyworm.com). Cultures of AV-T, AV-T Δ*1132*, and AV-T Δ*1132*/Tn7-*1132* were grown in 2 mL LB from pure frozen stocks to OD_600_ ~ 0.5 (shaking, 37°C). 5 µL of a LB control, AV-T, AV-T Δ*1132*, or AV-T Δ*1132*/Tn7-*1132* was injected into the hemolymph of a larvae of mass 150-200 mg (n=30 per condition; approximately 1.2 x 10^6^ CFU were administered per strain). Larvae were then housed in petri dishes in a humidified incubator at 37°C for 5 days. Each day, dead larvae were removed and surviving larvae counted.

### Mouse Virulence Assays

Approximately 1 × 10^8^ CFU were administered per mouse for infections to quantify the bacterial load. For mouse infections, overnight standing bacterial cultures at room temperature were sub-cultured in LB broth and grown at 37°C with shaking to an OD600 ~0.15, washed and re-suspended in PBS. Each mouse was inoculated intranasally with 50 µL of bacteria. Mice were anesthetized with isoflurane immediately prior to intranasal inoculation. At 24 hours, the mice were sacrificed and the lungs were harvested, homogenized, and ten-fold serial dilutions plated for CFU on 0.5X LB plates. The mouse strain used was C57BL/6J (females at 8-10 weeks of age) from Jackson Laboratories (JAX stock #000664). Experiments were carried out under the Institutional Animal Care and Use Committee guidelines.

### Statistics

Statistics were performed using GraphPad Prism 9.2.0 software for Windows (GraphPad Software, San Diego, California USA, www.graphpad.com). The following statistical tests were utilized: (1) Mann-Whitney test for the mouse experiments (two-tailed), two-sample switching assays (two-tailed), motility assays (two-tailed) and qRT-PCR experiments (two-tailed or multiple, as indicated); (2) Log-Rank (Mantel-Cox) test for the *Galleria mellonella* experiments; (3) Welch’s ANOVA analysis for three-sample switching assays; and (4) Student’s two-tailed *t* test for capsule width measurements from the TEM micrographs. The Mann-Whitney test was utilized instead of a Student’s *t* test to allow for the possibility or reality of a non-Gaussian distribution. Welch’s ANOVA was chosen over standard ANOVA to allow for unequal variance between samples.

## Data Availability Statement

The RNA-Seq data presented in this study have been deposited in NCBI’s Gene Expression Omnibus and are accessible through GEO Series accession number GSE185730 (https://www.ncbi.nlm.nih.gov/geo/query/acc.cgi?acc=GSE185730).

## Ethics Statement

The animal study was reviewed and approved by Emory University Institutional Animal Care and Use Committee.

## Author Contributions

PR and AT conceptualized and designed the study and experiments. AT carried out all of the experiments except the RNA sequencing, mouse experiments, and electron microscopy. CC performed the mouse experiments and subsequent data analysis. AT analysed all other data with guidance from PR. AT and CC wrote the original manuscript. All authors contributed to the article and approved the submitted version.

## Funding

Research in the PNR Laboratory is supported by T32 AI106699 to AT and NIH R01 AI72219 and R21 AI115183 and Department of Veterans Affairs awards I01 BX001725 and IK6BX004470 to PR. Research in the DSW Laboratory is supported by a Department of Veteran’s Affairs award BX002788. DW is also supported by a Burroughs Wellcome Fund Investigators in the Pathogenesis of Infectious Disease award. The content expressed herein is solely the responsibility of the authors and does not necessarily represent the official views of the NIH or the Department of Veterans Affairs.

## Conflict of Interest

The authors declare that the research was conducted in the absence of any commercial or financial relationships that could be construed as a potential conflict of interest.

## Publisher’s Note

All claims expressed in this article are solely those of the authors and do not necessarily represent those of their affiliated organizations, or those of the publisher, the editors and the reviewers. Any product that may be evaluated in this article, or claim that may be made by its manufacturer, is not guaranteed or endorsed by the publisher.

## Supplementary Material

Supplementary Figure 1 Deletion of *1132* does not affect *abaI* expression. qRT-PCR experiments comparing wild-type VIR-O and VIR-O Δ*1132* indicate no significant difference in the expression of *abaI*, using *16S* as an internal control (two-tailed Mann-Whitney test, ns, not significant). Results are the average of three biological replicates.

Supplementary Figure 2 VIR-O Δ*1132* cells contain quorum sensing signal (AHL), but do not secrete it. Cultures of wild-type VIR-O, VIR-O Δ*1132*, and VIR-O Δ*abaI* were grown to the same OD_600_ and plated as a line onto a soft-agar lawn containing *Agrobacterium tumefaciens* (*traG*::*lacZ*) and X-Gal. 10% SDS was added to a well at the center of the plate, lysing the cells.

Supplementary Figure 3 AV-T Δ*1132* switches to the VIR-O variant at the same frequency as wild-type AV-T. Bars represent averages of six colonies assayed for switching frequency at 18 hours of growth. Error bars indicate standard deviation of the mean, and there is no statistical significance as assessed by Welch’s ANOVA.

Supplementary Figure 4 K locus genes for capsule polysaccharide synthesis and export are not transcriptionally regulated by 1132. Bars for AV-T Δ*1132* and AV-T Δ*1132*/Tn7-*1132* represent averages of three biological replicates. Error bars indicate standard deviation of the mean, and there is no statistical significance in expression across strains as assessed by multiple Mann-Whitney tests.

Supplementary Figure 5
**(A)** Growth curves for VIR-O, AV-T, VIR-O Δ*1132*, and AV-T Δ*1132* in LB media. Data represents two biological replicates, and error bars indicate standard deviation of the mean. **(B)** *Galleria mellonella* infected with VIR-O Δ*1132* show no significant difference in mortality compared with wild-type VIR-O (n=30 per strain) as assessed by a log-rank (Mantel-Cox) test. **(C)** Mice were infected intranasally with either VIR-O or VIR-O Δ*1132*. At 24 hours post-inoculation, bacteria were recovered from the lungs, spleen, and liver tissues and CFU/g quantified. There is no significant difference between the two strains in all tissues as assessed by a Mann-Whitney test. ns, not significant.
