# Supplementary material for: A LysR-Type Transcriptional Regulator Controls Multiple Phenotypes in Acinetobacter baumannii
Source: Front Cell Infect Microbiol. 2021 Nov 4;11:778331. doi: 10.3389/fcimb.2021.778331 (PMC8601201; doi:10.3389/fcimb.2021.778331)
Supplement: Supplementary Figure 1 — Deletion of 1132 does not affect abaI expression. qRT-PCR experiments comparing wild-type VIR-O and VIR-O Δ1132 indicate no significant difference in the expression of abaI, using 16S as an internal control (two-tailed Mann-Whitney test, ns, not significant). Results are the average of three biological replicates. [file Presentation_1.pptx]

## Slide 1
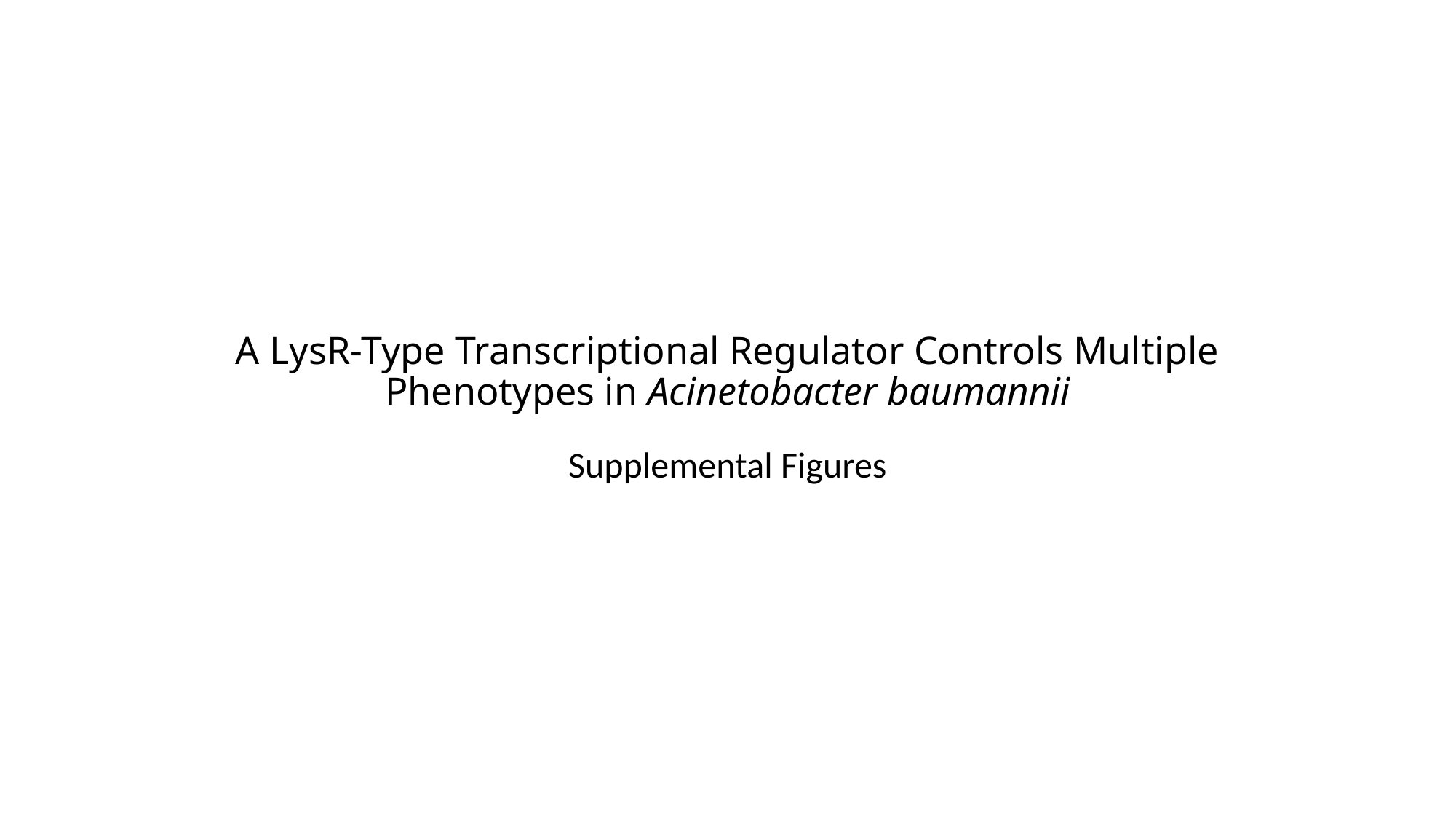

# A LysR-Type Transcriptional Regulator Controls Multiple Phenotypes in Acinetobacter baumannii
Supplemental Figures

## Slide 2
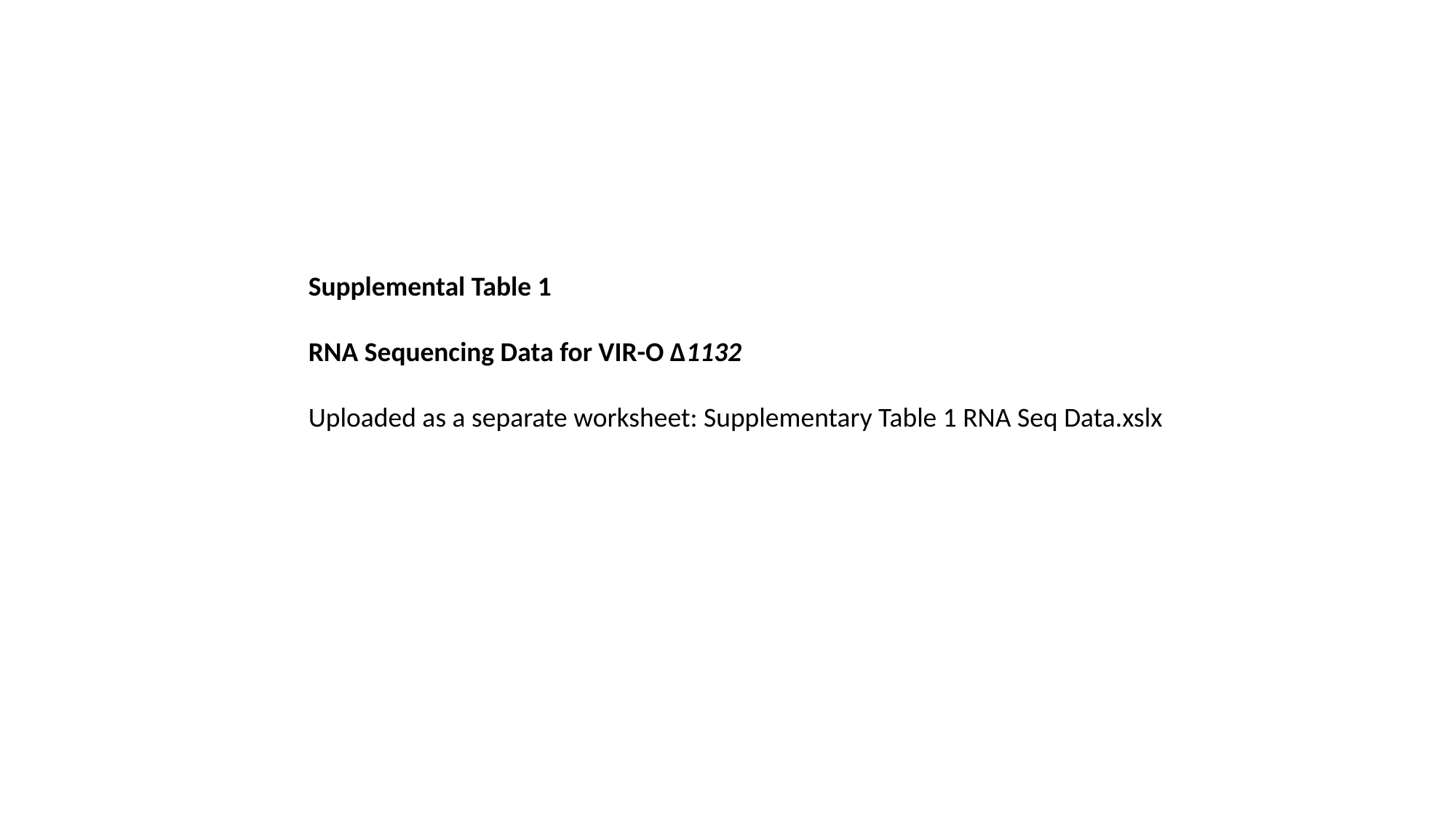

Supplemental Table 1
RNA Sequencing Data for VIR-O Δ1132
Uploaded as a separate worksheet: Supplementary Table 1 RNA Seq Data.xslx

## Slide 3
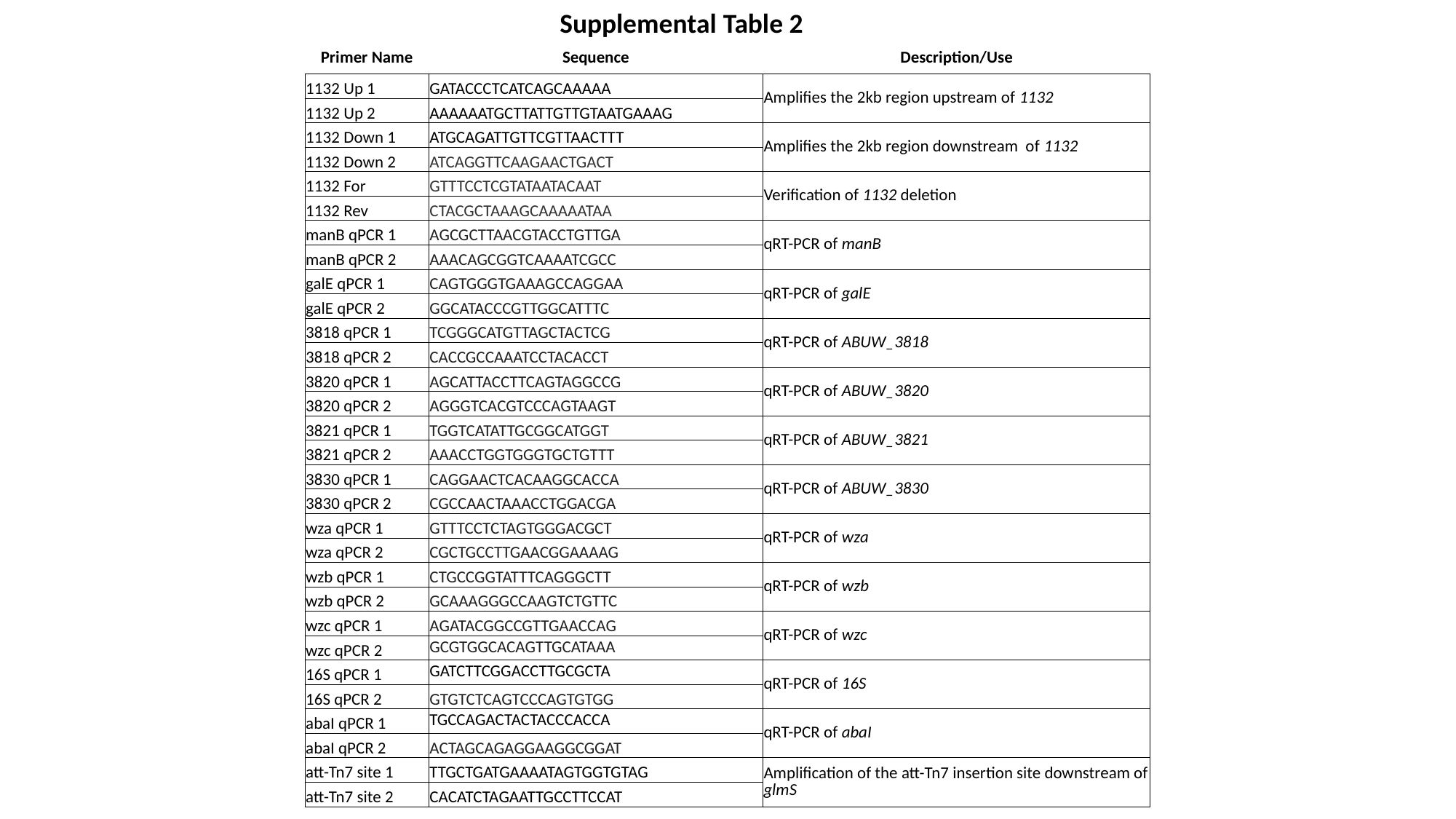

Supplemental Table 2
| Primer Name | Sequence | Description/Use |
| --- | --- | --- |
| 1132 Up 1 | GATACCCTCATCAGCAAAAA | Amplifies the 2kb region upstream of 1132 |
| 1132 Up 2 | AAAAAATGCTTATTGTTGTAATGAAAG | |
| 1132 Down 1 | ATGCAGATTGTTCGTTAACTTT | Amplifies the 2kb region downstream of 1132 |
| 1132 Down 2 | ATCAGGTTCAAGAACTGACT | |
| 1132 For | GTTTCCTCGTATAATACAAT | Verification of 1132 deletion |
| 1132 Rev | CTACGCTAAAGCAAAAATAA | |
| manB qPCR 1 | AGCGCTTAACGTACCTGTTGA | qRT-PCR of manB |
| manB qPCR 2 | AAACAGCGGTCAAAATCGCC | |
| galE qPCR 1 | CAGTGGGTGAAAGCCAGGAA | qRT-PCR of galE |
| galE qPCR 2 | GGCATACCCGTTGGCATTTC | |
| 3818 qPCR 1 | TCGGGCATGTTAGCTACTCG | qRT-PCR of ABUW\_3818 |
| 3818 qPCR 2 | CACCGCCAAATCCTACACCT | |
| 3820 qPCR 1 | AGCATTACCTTCAGTAGGCCG | qRT-PCR of ABUW\_3820 |
| 3820 qPCR 2 | AGGGTCACGTCCCAGTAAGT | |
| 3821 qPCR 1 | TGGTCATATTGCGGCATGGT | qRT-PCR of ABUW\_3821 |
| 3821 qPCR 2 | AAACCTGGTGGGTGCTGTTT | |
| 3830 qPCR 1 | CAGGAACTCACAAGGCACCA | qRT-PCR of ABUW\_3830 |
| 3830 qPCR 2 | CGCCAACTAAACCTGGACGA | |
| wza qPCR 1 | GTTTCCTCTAGTGGGACGCT | qRT-PCR of wza |
| wza qPCR 2 | CGCTGCCTTGAACGGAAAAG | |
| wzb qPCR 1 | CTGCCGGTATTTCAGGGCTT | qRT-PCR of wzb |
| wzb qPCR 2 | GCAAAGGGCCAAGTCTGTTC | |
| wzc qPCR 1 | AGATACGGCCGTTGAACCAG | qRT-PCR of wzc |
| wzc qPCR 2 | GCGTGGCACAGTTGCATAAA | |
| 16S qPCR 1 | GATCTTCGGACCTTGCGCTA | qRT-PCR of 16S |
| 16S qPCR 2 | GTGTCTCAGTCCCAGTGTGG | |
| abaI qPCR 1 | TGCCAGACTACTACCCACCA | qRT-PCR of abaI |
| abaI qPCR 2 | ACTAGCAGAGGAAGGCGGAT | |
| att-Tn7 site 1 | TTGCTGATGAAAATAGTGGTGTAG | Amplification of the att-Tn7 insertion site downstream of glmS |
| att-Tn7 site 2 | CACATCTAGAATTGCCTTCCAT | |

## Slide 4
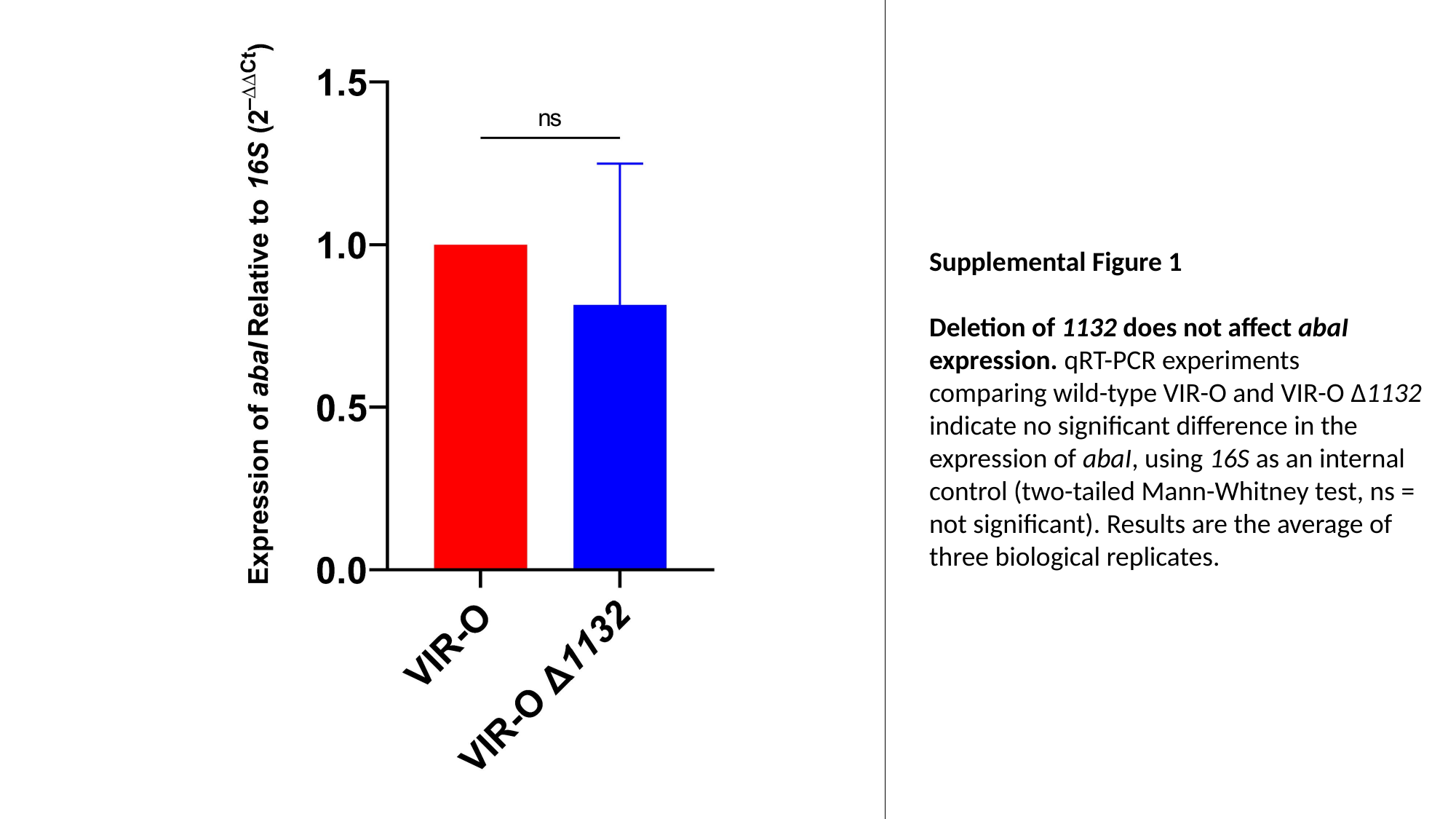

Supplemental Figure 1
Deletion of 1132 does not affect abaI expression. qRT-PCR experiments comparing wild-type VIR-O and VIR-O Δ1132 indicate no significant difference in the expression of abaI, using 16S as an internal control (two-tailed Mann-Whitney test, ns = not significant). Results are the average of three biological replicates.

## Slide 5
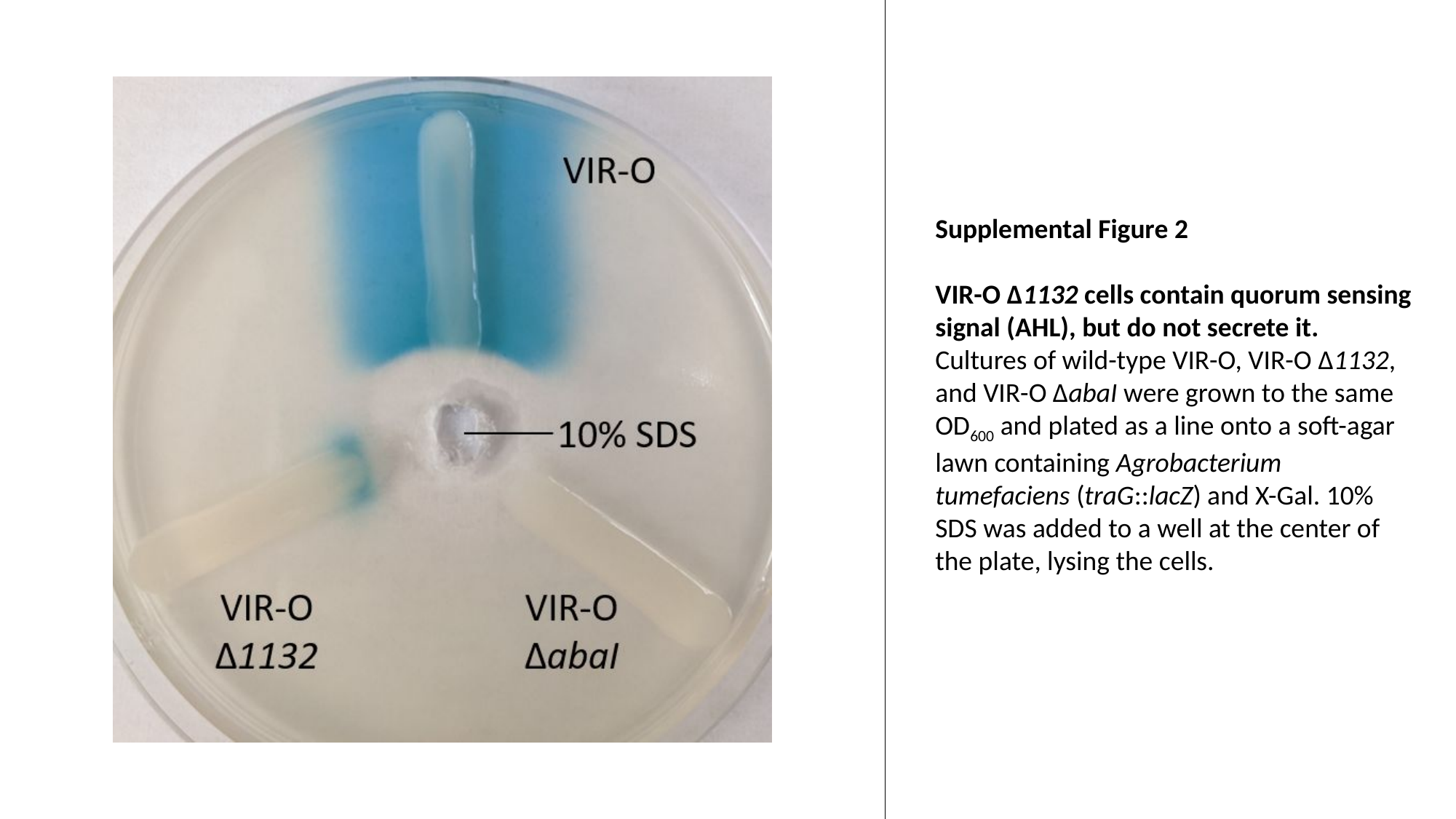

Supplemental Figure 2
VIR-O Δ1132 cells contain quorum sensing signal (AHL), but do not secrete it. Cultures of wild-type VIR-O, VIR-O Δ1132, and VIR-O ΔabaI were grown to the same OD600 and plated as a line onto a soft-agar lawn containing Agrobacterium tumefaciens (traG::lacZ) and X-Gal. 10% SDS was added to a well at the center of the plate, lysing the cells.

## Slide 6
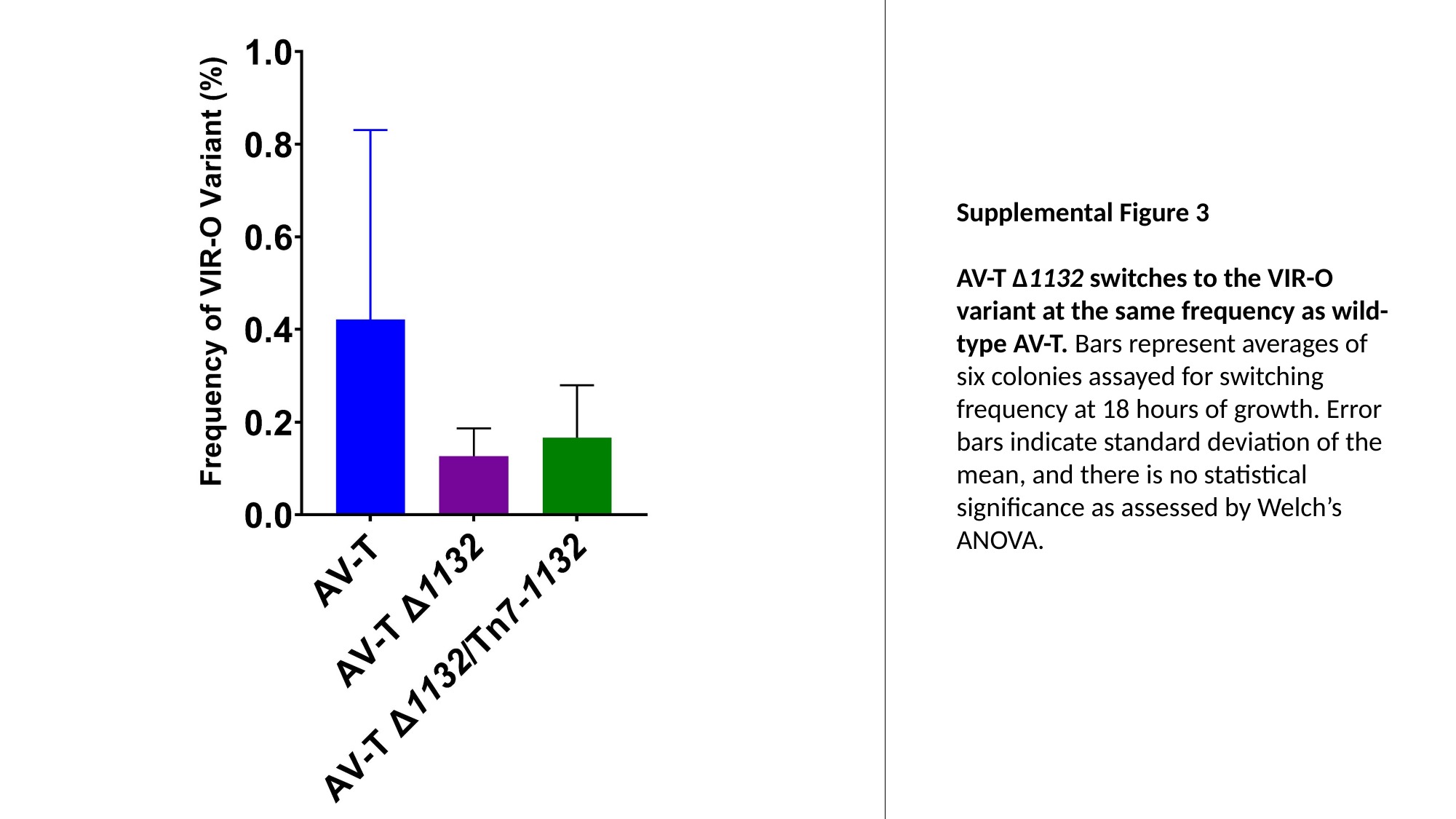

Supplemental Figure 3
AV-T Δ1132 switches to the VIR-O variant at the same frequency as wild-type AV-T. Bars represent averages of six colonies assayed for switching frequency at 18 hours of growth. Error bars indicate standard deviation of the mean, and there is no statistical significance as assessed by Welch’s ANOVA.

## Slide 7
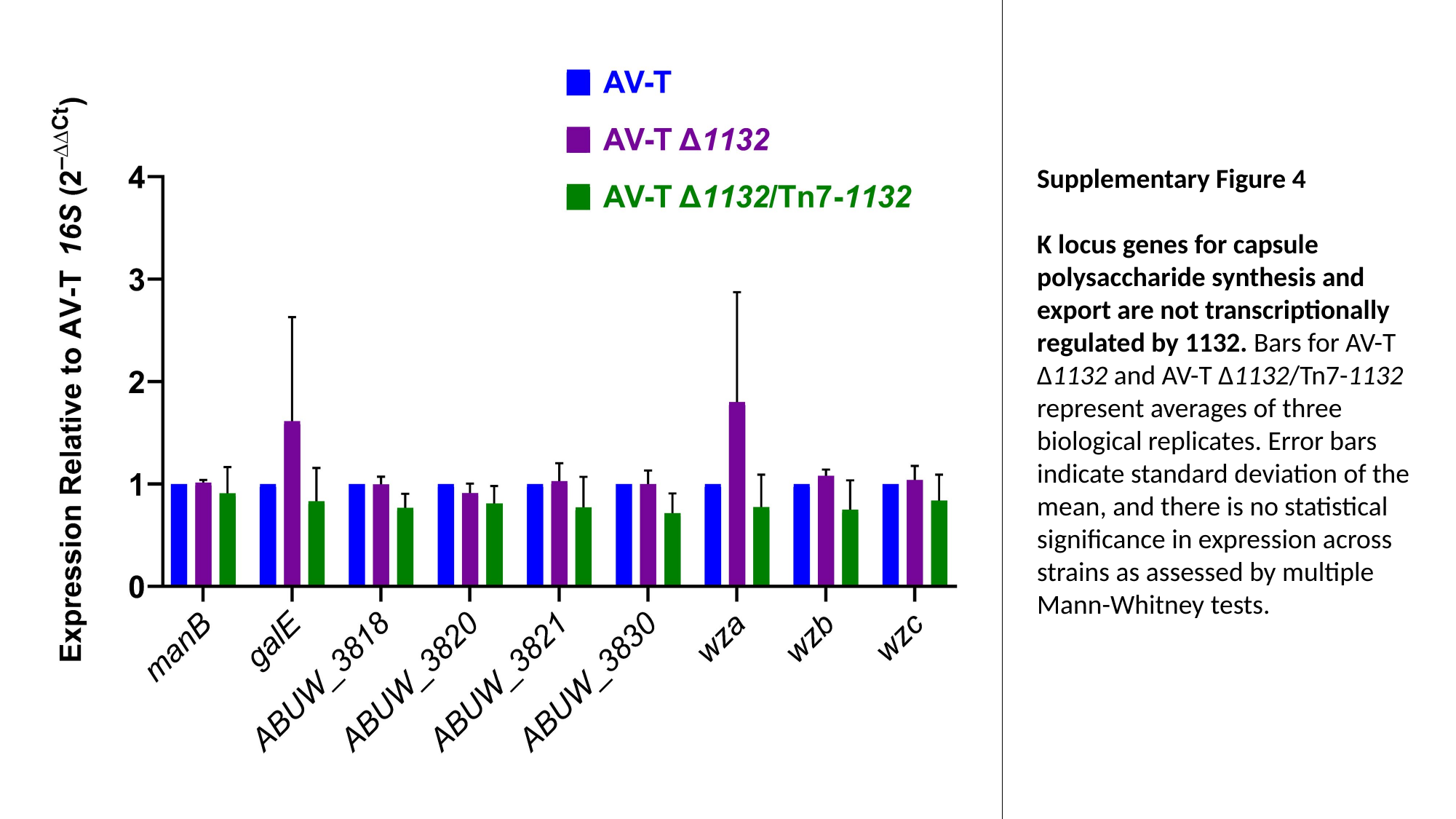

Supplementary Figure 4
K locus genes for capsule polysaccharide synthesis and export are not transcriptionally regulated by 1132. Bars for AV-T Δ1132 and AV-T Δ1132/Tn7-1132 represent averages of three biological replicates. Error bars indicate standard deviation of the mean, and there is no statistical significance in expression across strains as assessed by multiple Mann-Whitney tests.

## Slide 8
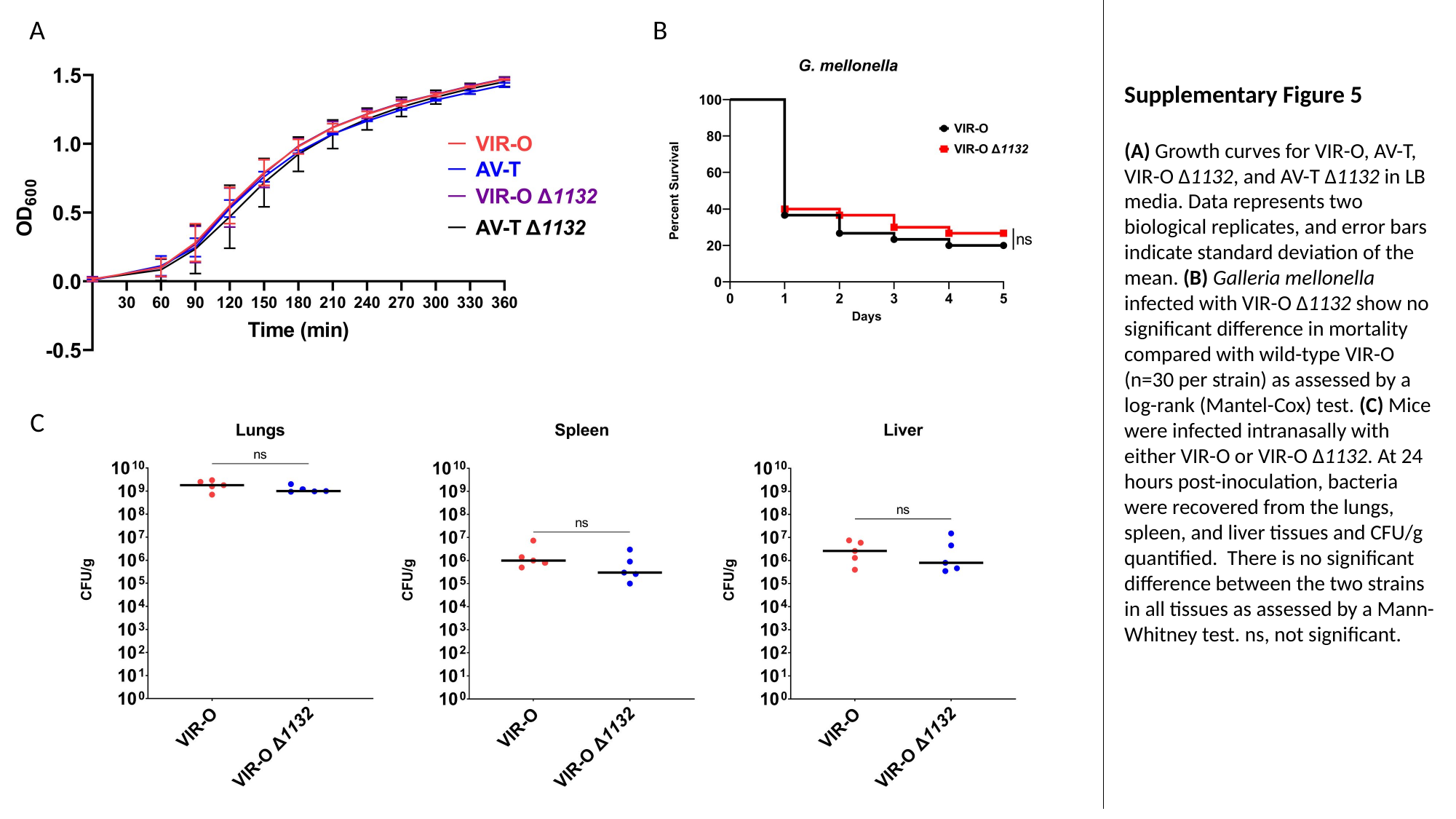

A
B
Supplementary Figure 5
(A) Growth curves for VIR-O, AV-T, VIR-O Δ1132, and AV-T Δ1132 in LB media. Data represents two biological replicates, and error bars indicate standard deviation of the mean. (B) Galleria mellonella infected with VIR-O Δ1132 show no significant difference in mortality compared with wild-type VIR-O (n=30 per strain) as assessed by a log-rank (Mantel-Cox) test. (C) Mice were infected intranasally with either VIR-O or VIR-O Δ1132. At 24 hours post-inoculation, bacteria were recovered from the lungs, spleen, and liver tissues and CFU/g quantified. There is no significant difference between the two strains in all tissues as assessed by a Mann-Whitney test. ns, not significant.
C
